# A Service-Oriented Real-Time Communication Scheme for AUTOSAR Adaptive Using OPC UA and Time-Sensitive Networking

**DOI:** 10.3390/s21072337

**Published:** 2021-03-27

**Authors:** Anna Arestova, Maximilian Martin, Kai-Steffen Jens Hielscher, Reinhard German

**Affiliations:** 1 Computer Science 7, Computer Networks and Communication Systems, University of Erlangen-Nürnberg, 91058 Erlangen, Germany; kai-steffen.hielscher@fau.de (K.-S.J.H.); reinhard.german@fau.de (R.G.); 2 Siemens Mobility GmbH, 91058 Erlangen, Germany; maximilian.martin@siemens.com

**Keywords:** AUTOSAR adaptive, OPC UA, time-sensitive networking, vehicle communication

## Abstract

The transportation industry is facing major challenges that come along with innovative trends like autonomous driving. Due to the growing amount of network participants, smart sensors, and mixed-critical data, scalability and interoperability have become key factors of cost-efficient vehicle engineering. One solution to overcome these challenges is the AUTOSAR Adaptive software platform. Its service-oriented communication methodology allows a standardized data exchange that is not bound to a specific middleware protocol. OPC UA is a communication standard that is well-established in modern industrial automation. In addition to its Client–Server communication pattern, the newly released Publish–Subscribe (PubSub) architecture promotes scalability. PubSub is designed to work in conjunction with Time-Sensitive Networking (TSN), a collection of standards that add real-time aspects to standard Ethernet networks. TSN allows services with different requirements to share a single physical network. In this paper, we specify an integration approach of AUTOSAR Adaptive, OPC UA, and TSN. It combines the benefits of these three technologies to provide deterministic high-speed communication. Our main contribution is the architecture for the binding between Adaptive Platform and OPC UA. With a prototypical implementation, we prove that a combination of OPC UA Client–Server and PubSub qualifies as a middleware solution for service-oriented communication in AUTOSAR.

## 1. Introduction

The automotive industry is rushing towards new trends like autonomous driving and Car2X [1,2,3]. Vehicles are becoming increasingly intelligent and interconnected with the outside world and with each other [4]. The number of new functions in a vehicle is growing rapidly. Particularly in the context of autonomous driving, the need for special sensors that perceive the environment and complex sensor data fusion functions has increased. In addition to the actual control of the vehicle, other components such as driving assistants and infotainment gain in significance. As a consequence, the amount of mixed-critical data to be processed and exchanged has grown enormously.

The current state of the art is that each functionality is implemented on an adapted Electronic Control Unit (ECU). The ECUs are interconnected via proprietary communication buses like CAN and FlexRay [4]. The rapid growth of data and interconnected functions encourage the automotive industry to look for more adaptive and high-end performance software platforms, as well as communication technologies that are able to achieve high throughput, provide determinism, and keep up with the dynamic communication pattern of new features.

The Automotive Open System Architecture (AUTOSAR) (https://www.autosar.org, last accessed on 1 March 2021) is a global development partnership that addresses these software platform requirements with the ongoing development of the new standard AUTOSAR Adaptive Platform (AP). The new platform offers a high level of flexibility regarding software allocation, the underlying hardware, and network communication. AP relies on the dynamic deployment of customer applications and the use of highly parallelized processors. In comparison, the previous AUTOSAR standard, now called the AUTOSAR Classic Platform (CP), mainly encounters the requirements of deeply embedded systems [5]. AUTOSAR Adaptive is designed to provide sufficient capacities for the processing of complex functions, e.g., image processing in Advanced Driver Assistance Systems (ADAS) and Automated Driving Functions (ADF), and to support distributed systems.

Moreover, AP is designed to fully support Ethernet-based communication. To better support automotive use cases, AUTOSAR specified the service-oriented communication middleware SOME/IP (Scalable service-Oriented Middleware over Internet Protocol) for the automotive/embedded field [6] and especially for AP. Some contents of SOME/IP (events, fields, etc.) are even integrated in AP. Therefore, the Communication Management (CM) [2] of AP already includes standardized bindings for SOME/IP, but also for the data-centric middleware protocol Data Distribution Service (DDS) [7]. Besides the direct binding of a middleware protocol to CM, gateway solutions represent a further solution for the binding of additional middleware solutions [8,9]. At the moment, (hard) real-time capable solutions exist neither with SOME/IP nor with DDS. Nevertheless, AP allows the specification of other direct middleware bindings, e.g., for OPC UA. The OPC UA technology supports real-time communication patterns. For a holistic real-time solution, a deterministic network technology like TSN is required on Open Systems Interconnection (OSI) layers 1 and 2.

The Open Platform Communications Unified Architecture (OPC UA) is a machine-to-machine (M2M) communication technology that is established in modern industrial automation. It is based on a service-oriented architecture (SOA). The focus of OPC UA is flexible communication using a Client–Server (C/S) and a Publish–Subscribe (PubSub) communication pattern to connect machines from different vendors. OPC UA satisfies the vertical communication covering all levels of the automation pyramid including field, control, supervisory, planning, and management level [10]. Horizontal communication based on OPC UA is currently only established for controller-to-controller communication. In addition, OPC UA is seen as the key communication protocol in Industry 4.0 and IIoT (Industrial Internet of Things) [11]. Additionally, OPC UA empowers connectivity to cloud solutions [12] and is gaining popularity in smart grids [13].

In 2018, the OPC Foundation started the Field Level Communications (FLC) initiative. Experts from global players like Cisco, Intel, Siemens, and TTTech join forces to establish OPC UA on the field level. This shall enable unified and vendor-independent communication among devices (sensors and actuators) and between field devices, controllers, and the cloud [14]. Today, fieldbuses like PROFIBUS and Modbus as well as Ethernet-based protocols like PROFINET and EtherCat can be found on the field level [12]. The FLC initiative aims to equip OPC UA with features required for industrial use cases (e.g., determinism, safety, security). Real-time capability is achieved with the OPC UA PubSub communication pattern in conjunction with Time-Sensitive Networking (TSN). In addition, the FLC initiative is developing methods for the configuration of Quality of Service (QoS) parameters, including TSN parameters [14].

The Institute of Electrical and Electronics Engineers (IEEE) Time-Sensitive Networking Task Group (https://1.ieee802.org/tsn/, last accessed on 28 February 2021) provides extensions to Ethernet networks defined in IEEE 802.1 and IEEE 802.3 to enable deterministic and reliable transmission of time-sensitive network traffic. TSN allows for mix time-critical network traffic with best-effort services. This so-called network convergence is particularly beneficial for the implementation of mixed-critical systems. The specification of a number of TSN standards has been completed while others are still in progress. The TSN Task Group has initiated several projects to define TSN profiles for different application domains such as industry, automotive, and aerospace. TSN is an upcoming technology which is only used sporadically today. One of the reasons is that there are not many TSN-capable devices on the market yet and that only few TSN mechanisms are supported. These mechanisms consist primarily of time synchronization and time-triggered transmission of data packets.

The combination of TSN and OPC UA enables flexible and deterministic communication for a wide range of devices from different manufacturers lowering the configuration overhead. Thus, it is not only interesting for industrial automation [10,12,15,16,17], but also for other application domains like the automotive [18,19] and railway industry [20].

**Figure 1:**
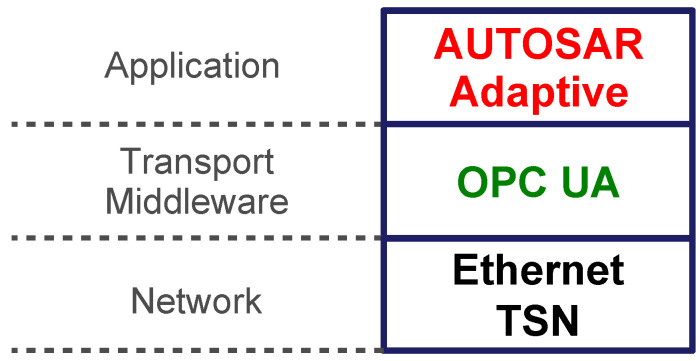
Integration stack of AUTOSAR Adaptive, Open Platform Communications Unified Architecture (OPC UA), and Time-Sensitive Networking (TSN).

By integrating the AUTOSAR Adaptive Platform, OPC UA, and TSN, as shown in Figure 1, the benefits of these technologies can be combined. This solution addresses the abovementioned challenges of the automotive industry. The goal of this paper is to show how these three technologies can be integrated and how determinism can be achieved. We give an overview of the mentioned technologies in Section 2. In Section 3, we review the related work. We present the integration architecture and discuss the impact of architectural decisions including crucial aspects of the implementation in Section 4. Furthermore, we describe an experimental evaluation of the proposed communication architecture and discuss the results in Section 7. Finally, we conclude our work and give an insight into future activities in Section 9.

## 2. Fundamentals

As Figure 1 illustrates, our intention is to provide an OPC UA network binding for service-oriented communication in AUTOSAR Adaptive. In this scenario, the transmission of the OPC UA messages shall take place over TSN. In the following, we introduce the three components in more detail.

### 2.1. AUTOSAR Adaptive

AUTOSAR is a development partnership of players in the automotive sector. Partners join forces to develop standardized system functions and functional interfaces for ECUs. The AUTOSAR Classic Platform is a well-established standard for deeply embedded ECUs. However, the CP is not fit for highly parallelized and distributed systems as needed for innovative trends like autonomous driving. The AUTOSAR Adaptive Platform is an emerging standard that addresses these shortcomings. It is an execution environment that builds upon any operating system compliant with the Portable Operating System Interface (POSIX) standard [5].

**Figure 2:**
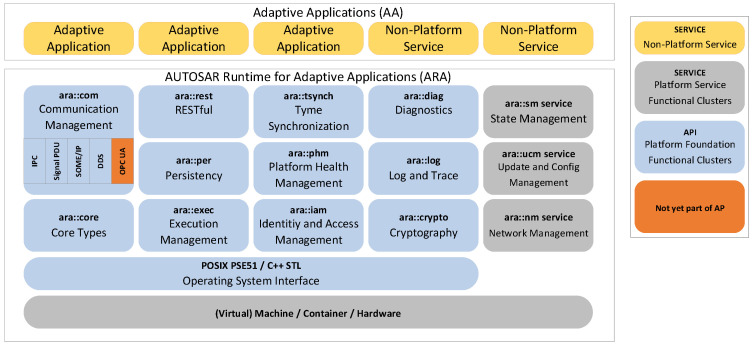
Logical view of Adaptive Platform (AP) architecture including the OPC UA binding, see ([5] Figure 3–1).

The logical view of the AP architecture is shown in Figure 2. Adaptive Applications (AAs) run on top of the AUTOSAR Runtime for Adaptive Applications (ARA) [5]. ARA is organized in so-called functional clusters. The functional clusters either belong to the Adaptive Platform Foundation or Adaptive Platform Services [5]. Clusters provide C++ interfaces for access to the AUTOSAR runtime. In addition, any Adaptive Application can be designed to provide services to other Adaptive Applications. In order to give a better insight into our work, we take a closer look at the Communication Management (CM), the Execution Management (EM), and the service-oriented approach of AP in general.

#### 2.1.1. Communication Management

The Communication Management [2], also known as ara::com, implements the service-oriented architecture pattern to achieve flexibility and scalability for distributed processing. A service is offered by a provider and used by a consumer. Services consist of:

- Events that represent read-only notifications
- Methods facilitating remote procedure calls
- Fields enabling read and write access to data points

AP allows for perform service-oriented communication over different middleware protocols and interprocess communication (IPC). Network bindings already exist for SOME/IP, DDS, and signal-based communication (Signal PDU) [5].

AUTOSAR’s service-oriented communication offers a dynamic establishment of communication paths. The central component for runtime discovery is the service registry. Applications that provide services register them with the service registry. Service-consuming applications can find services by querying the registry. After that, they can call services directly at the provider. This process is called service discovery. Similar to service access, discovery calls are mapped to the specific middleware protocol as well.

#### 2.1.2. Execution Management

The Execution Management [21] is responsible for managing the execution of the platform and user applications. Among other things, it is in charge of the initialization of the platform as well as the startup and shutdown of applications. It configures the platform and applications according to a manifest, the Execution Manifest. AUTOSAR manifests are XML-based files describing services, applications, the underlying machines, and their configuration [5]. EM works in conjunction with the operating system (OS). One important aspect of the cooperation between the Execution Manager and the OS is the configuration of scheduling policies. Thus, more critical applications can be scheduled to run with a higher priority [21].

#### 2.1.3. Example: Service Interface Realization

**Figure 3:**
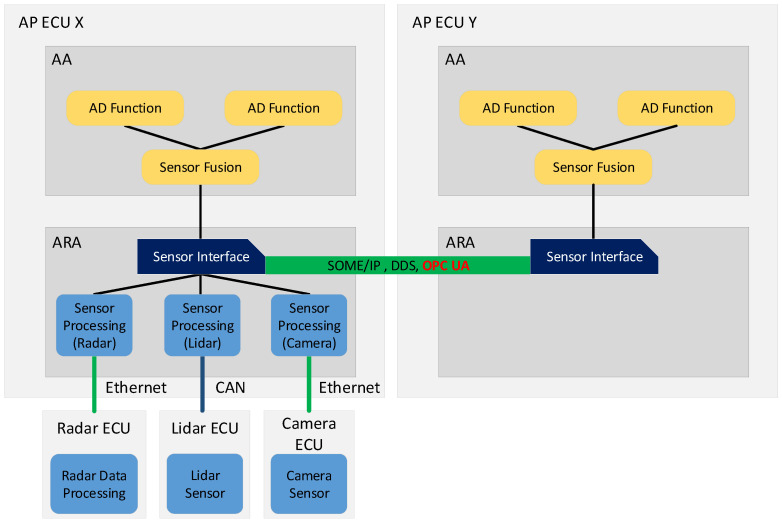
Realization of sensor interfaces with AP, see ([22] Figure 4.3).

The service-oriented approach of AP allows custom applications in AA and platform applications in ARA to expose data and methods to other applications on the same or on a different device. This procedure promotes flexible software engineering and the definition of proper interfaces. In addition, the acquisition of sensor data can be realized in a service-oriented and generic manner. In [22], the authors propose to realize a sensor interface as a service in AP to simplify the access to different sensor data and to support the sensor data fusion function. As shown in Figure 3, one AP instance that is running on ECU X is connected to ECUs integrating different sensors over different communication technologies. The adaptive applications running on the platform perform sensor data fusion for automated driving (AD) functions. Another AP instance (running on ECU Y) depends on the sensor data. By providing a sensor interface, the AP on ECU X can exchange the sensor data with AP on ECU Y over a service-oriented middleware like SOME/IP, DDS, or OPC UA. This exchange can be realized in AP cyclically or on demand. Additionally, the design of the sensor interface as a service enables a simplified connection of new sensors.

In this paper, we focus on providing a middleware binding solution to enable such a service interface-based data exchange over OPC UA.

### 2.2. OPC UA

OPC UA is an open M2M communication standard developed by the OPC Foundation (https://opcfoundation.org, last accessed on 1 March 2021) that is established in modern industry automation. It provides a manufacturer-independent and service-oriented architecture. OPC UA’s goal is to provide universal data exchange over different levels (“from the sensor to the cloud”). OPC UA features a rich semantic model, so-called information model, as well as a variety of transport protocol mappings and communication patterns. It additionally provides specifications for, amongst others, security [23], diagnosis and auditing [24], and safety [25], which forms a good basis for future vehicle platforms. In the following, we go into more detail about the communication and information model.

#### 2.2.1. Communication Model

**Figure 4:**
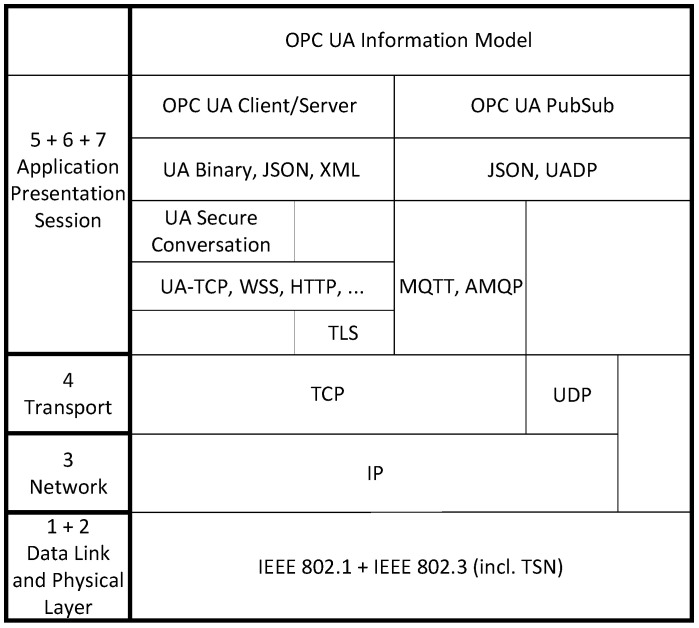
OPC UA in the Open Systems Interconnection (OSI) reference model.

OPC UA offers two communication models: C/S and PubSub. The OPC UA C/S scheme implements a classic connection-oriented one-to-one communication pattern. A server acts as an information source while a client consumes information. As shown in Figure 4, C/S supports different message mappings (Binary, JSON, XML) and transport protocol mappings (HTTP(S), UA-TCP, etc.). In the first place, the C/S scheme enables reliable communication between two parties. However, C/S is not designed to be real time capable. The C/S pattern was recently complemented by PubSub communication [26]. PubSub decouples publishers and subscribers. This means that publishers are able to perform connectionless many-to-many communication. PubSub supports different message mappings and transport protocol mappings as well. While PubSub also allows human-readable message mapping formats such as JSON, we decided to use the binary message mapping UA Datagram Protocol (UADP) due to its efficient serialization. PubSub supports payload transport over Message Queuing Telemetry Transport (MQTT), Advanced Message Queuing Protocol (AMQP), User Datagram Protocol (UDP), and raw Ethernet. In case of the UDP and raw Ethernet mapping, subscribers register to the multicast IP or MAC address associated with the data of interest. In contrast to C/S, PubSub over UDP or raw Ethernet has the potential for deterministic data transmission when used in conjunction with Time-Sensitive Networking as both have a connectionless nature. We chose the UDP transport protocol mapping in our binding approach because it supports deterministic transmission and it allows for exploiting IP-based multicast mechanisms.

#### 2.2.2. Address Space and Information Model

**Figure 5:**
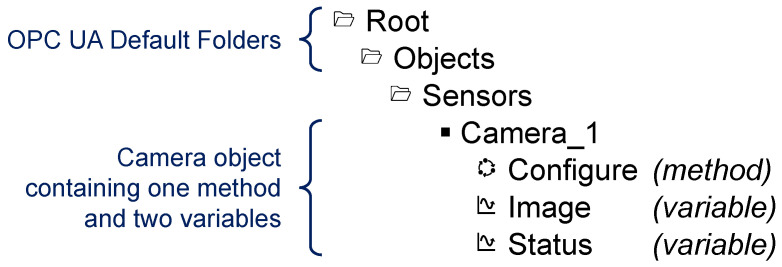
Exemplary information model for a sensor interface.

The OPC UA information model is an organizational framework that represents the information resources of a system. Complex relationships can be modeled using object-oriented concepts. It is based on the modeling methodology of the address space model specified in [27]. The address space is an information collection that a server provides to its clients. Elements of this model are represented as nodes. The specification of the information model [28] describes standardized nodes of a server address space. These are the nodes that a blank server contains. Furthermore, custom elements and types can be defined. An exemplary information model is shown in Figure 5. Moreover, OPC UA provides methods to perform simple or complex processing of the modeled data in the address space, e.g., by applying interpolation functions or time series analysis.

Each node is an instance of a node class. Node classes define specific attributes that describe the nodes and references that define their relationship to other nodes. OPC UA defines eight node classes. For example, nodes derived from the node class Object may represent real-world objects like sensors (camera sensor in Figure 5) or whole systems. Instances of Variable node class are used to represent the content of an object, status and current image of the camera sensor. The Image value of the camera sensor may have the type ByteString. Nodes with executable functions are typed by the node class Method, see method Configure in Figure 5 [27].

The information model can be used as the central information resource for both C/S and PubSub. PubSub does not necessarily require an information model, nor is it dependent on C/S. However, the combination of PubSub and C/S through a shared information model may be advantageous. An OPC UA server may provide sensor interfaces as represented in Figure 5. Over the C/S communication pattern, OPC UA clients can read or write values. Using the same information model for the PubSub scheme, OPC UA capable devices can subscribe to a cyclic transmission of sensor values in a real time or best-effort scheme. Additionally, PubSub configuration settings can be represented within an information model (Configuration Model). Thus, the C/S communication scheme can be used to provide a simple and standardized way to configure PubSub communication.

### 2.3. Time-Sensitive Networking

Time-Sensitive Networking provides a set of standards that extend Ethernet by the following aspects, see [29]:

- Synchronization of time (802.1AS-2020, etc.)
- Reliability (802.1CB, 802.1Qci, etc.)
- Latency (802.1Qbv, 802.1Qcr, 802.1Qch, 802.1Qbu, etc.)
- Resource Management (802.1Qcc, 802.1Qat, etc.)

**Figure 6:**
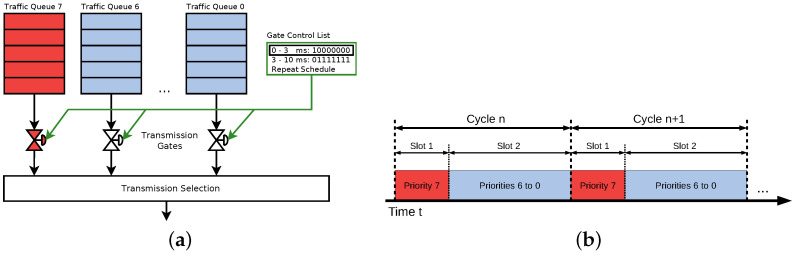
Methodology of Time-Aware Shaping (TAS). (**a**) shows the TAS components for transmission selection within an egress port. (**b**) shows the corresponding serialization of the network traffic on wire.

It allows network flows with different Quality of Service (QoS) requirements to share a single physical network. Our focus will be the Time-Aware Shaper (TAS) as specified in IEEE 802.1Qbv [30] as it provides explicit support for time-triggered transmission of packets. TAS components reside in egress ports of a switch or an end system device, see Figure 6a. Each egress queue is assigned a gate that opens and closes according to a schedule defined in a gate control list. The gates are controlled by the gate driver. As shown in Figure 6a, the gate associated with the egress queue for scheduled traffic (time- and safety-critical traffic) opens for 3 ms and remains closed for 7 ms within a cycle of 10 ms. Egress ports of switches typically have eight egress queues. This allows for mapping each value of the PCP field of the VLAN tag directly to one egress queue. Otherwise, an explicit mapping is required. One or several queues can be dedicated to scheduled traffic. Usually, only one queue is assigned for time- and safety-critical traffic [31,32]. Other egress queues share the remaining time window of the TAS cycle or further subdivide the cycle into separate transmission slots. The serialization of network traffic on wire according to the TAS configuration in Figure 6a is shown in Figure 6b.

Time-aware scheduling requires all network participants to have the same notion of time. In the context of TSN, it is achieved by time synchronization protocols such as the Precision Time Protocol (PTP) published in IEEE 1588 [33] or the PTP profile gPTP for TSN, specified in IEEE 802.1AS-2020 [34]. Furthermore, the IEEE 802.1 TSN working group defines the standard IEEE 802.1Qcc [35] to improve the administration of TSN networks, which also includes calculating and configuring TAS schedules for involved network and end-system devices.

## 3. Related Work

The main contribution of this paper is the architecture for the binding between Adaptive Platform and OPC UA. Furthermore, we examine TSN as link layer technology. More specifically, we are presenting a network binding that exposes AUTOSAR service components (events, methods, fields) with the methodology of OPC UA. To the best of our knowledge, we are the first to propose a direct binding of the aforementioned technologies. While implementations of OPC UA over TSN already exist and numerous works have been published on this subject, the usage of OPC UA over TSN for real-time communication in AUTOSAR Adaptive is a novel concept. In the following, we want to provide an insight into literature that addresses the current state of the AUTOSAR Adaptive communication model, the benefits of the combination of OPC UA and TSN in factory automation, and OPC UA considerations in the automotive industry.

Regarding future automotive software platforms, AUTOSAR Adaptive is considered as a key technology to tackle the challenges of autonomous driving and Car2X in [1,4]. The authors in [4] state that AUTOSAR Adaptive will facilitate the development of a standardized platform for connected cars due to its wide range of platform services. While these works concentrate on the applicability and suitability of AUTOSAR Adaptive as a future vehicle platform, our research initially focuses on the binding concept of AUTOSAR Adaptive, OPC UA, and TSN and the evaluation of this combination for the in-vehicle communication. However, we also address the advantages of AP.

On the communication level, AP covers service-oriented communication with SOME/IP and DDS over Ethernet at the moment, but there is still a lack of a real-time communication scheme. TSN is such an option located on layer 2 of the OSI reference model. Although less dominant in literature, the automotive industry considers TSN a future trend in vehicle communication [3,4,36]. The authors of [3] identify TSN as appropriate technology for future car technology as it is expected to reduce wiring efforts. They clarify that, after chassis and engine, the wiring harness of a car is ranked third in both cost and weight. We have chosen to integrate TSN in order to be able to provide deterministic end-to-end latency for real-time communication in AP. The aforementioned advantages favor the use of the technology.

In the industrial domain, TSN is represented in many works [8,10,11,12,15,16,17,32,37,38,39]. Since TSN is only represented at the lower layers of the OSI model, it is often considered together with a high-level protocol. The best-known representative in this case is OPC UA. Several publications regard the combination of TSN and OPC UA as future real-time communication candidate at the field level [10,11,15,16,32,37]. TSN offers a variety of mechanisms such as EEE 802.1Qbv, IEEE 802.1Qch, IEEE 802.1Qcr, and IEEE 802.1Qbu to guarantee timely response [37]. OPC UA, on the other hand, provides a connectionless and multicast-capable PubSub pattern and flexible configuration of data to be published [16]. Ref. [32] shows with example calculations that OPC UA over TSN outperforms current Ethernet-based fieldbus systems, especially because it allows the use of a Gigabit physical layer. In [16], the authors evaluated the performance of OPC UA over TSN using the open-source implementation open62541 of OPC UA that supports C/S and PubSub. Our implementation also uses the open62541 stack due to the real-time performance considerations discussed in the following. The authors of [16] showed that this PubSub implementation allows submillisecond publishing intervals with jitter in the nanosecond range. Others propose OPC UA PubSub for the delivery of TSN stream requirements to network components that are able to reserve and configure the necessary resources in TSN networks [37,38,39]. OPC UA offers a rich information model that simplifies the configuration process [39] and the discovery of publishers [37]. Our work concentrates on the real-time capability achieved through the combination of both technologies. The configuration aspect is a future topic that we are planning to address, as mentioned in Section 9.

To put the potential of OPC UA into context, researchers have compared the performance of open62541 with implementations of the middleware Robot Operating System (ROS), DDS, and MQTT in [40]. Their benchmarks show a performance advantage of open62541 over MQTT and ROS implementations and a similar performance compared to the eProsima FastRTPS DDS stack. The authors additionally highlight the rich information model of OPC UA.

OPC UA is also a technology that is researched by the automotive industry. The thesis [18] evaluates OPC UA for Car2X communication. The author concludes that OPC UA C/S brings considerable advantages for Car2X: It offers a suitable security model and facilitates interoperability. Furthermore, Ref. [9] proposes a gateway enabling interoperability between VSOMEIP (GENIVI’s SOME/IP stack (https://github.com/GENIVI/vsomeip, last accessed on 1 March 2021) and OPC UA applications to assure vehicle interoperability in the IIoT context. Such a gateway solution can also be applied to translate OPC UA C/S messages to SOME/IP Client–Server messages and vice versa in the AUTOSAR Adaptive context. The authors only worked with the Client–Server pattern and did not provide a direct binding of OPC UA concepts to SOME/IP or AUTOSAR Adaptive concepts, in contrast to our solution. In [8], the same authors extend their gateway solution by an OPC UA PubSub and VSOMEIP Notify–Subscribe gateway application. On the link layer, the authors use raw Ethernet. The gateway application was tested in a small setup in which one or several OPC UA modules communicated with a VSOMEIP module over a gateway node. All applications were distributed on separate devices. The evaluation showed that the end-to-end transmission latency did not exceed the exchange interval chosen in the range of 1 ms to 10 s. Our binding approach avoids the overhead of an intermediate application. It additionally embeds TSN as link layer and is able to reliably perform cyclic publishing at a 1 ms interval even in stress scenarios.

## 4. Materials and Methods

The following software and hardware components were necessary to implement the communication scheme described herein.

### 4.1. AUTOSAR Adaptive Platform Implementation and Tooling

The reference implementation and further AUTOSAR tooling is accessible for AUTOSAR members. In case of universities and non-profit organizations and within the scope of ATTENDEES membership, no annual administration fees have to be paid. We have used the AUTOSAR Adaptive reference implementation of the release 19-03.

### 4.2. OPC UA Implementation and Tooling

OPC UA provides a high variety of open- and closed-source stacks, tooling, and documentation (IEC 62541). We have used the open62541 stack that is available on GitHub (https://github.com/open62541/open62541, last accessed on 2 March 2021) for our binding approach.

### 4.3. TSN Mechanisms

To be able to use the TSN TAS mechanisms on end-system devices, either a TSN-capable network interface card like Kontron PCIE-0400-TSN should be used or the available network interface card should be adapted for the TSN queuing discipline TAPRIO (https://tsn.readthedocs.io/qdiscs.html, last accessed on 15 February 2021). Additionally, a high-precision time synchronization protocol like PTP, see the Linux PTP Project (http://linuxptp.sourceforge.net/, last accessed on 18 March 2021), should be installed on all communicating devices. When connecting end devices over a switch, the switch must support TAS and provide a time synchronization mechanism.

## 5. Binding Architecture

In the following subsections, we explain fundamental architectural concepts of our integration approach. We will go into detail about the mapping of AUTOSAR service components, types, configuration parameters, and service discovery to OPC UA methodology. Additionally, we discuss the approach for a binding implementation for real-time systems. The represented binding approach aims at combining the benefits from the adapted automotive platform AP, the rich information and communication model of OPC UA, and the real-time features of TSN.

### 5.1. Protocol Binding

Our concept integrates OPC UA C/S and PubSub inside a single binding. Both OPC UA facets provide access to a single information model for the AUTOSAR Adaptive services, see Section 5.3. This covers the use case of simultaneous C/S and PubSub communication in the same application. For example, PubSub is suitable for real-time communication, while C/S provides access for diagnostics, management, and configuration.

**Figure 7:**
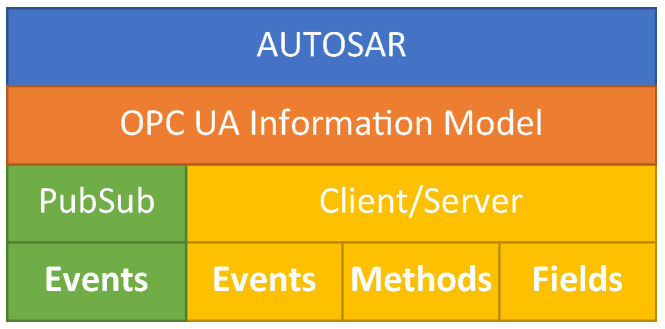
Overview of the combined OPC UA C/S and PubSub binding inside an AUTOSAR service provider.

Figure 7 shows that C/S and PubSub are integrated side by side in an AUTOSAR service provider. An OPC UA stack is placed inside the binding and contains the information model as well as the two protocol implementations. The information model is the shared data source for both protocols.

From the top-level perspective, the Adaptive Application provides data through mechanisms of the Adaptive Platform. The OPC UA binding inside the platform dispatches the data from the Adaptive Application into the information model, so that it is accessible via OPC UA C/S and PubSub.

From the bottom-level perspective, all service components (events, methods, fields) are accessible through C/S. PubSub is currently only used to transmit event data because PubSub does not match the concepts of methods and fields. The representation of the service components in OPC UA is described hereinafter.

### 5.2. Service Component Mapping

The three service components (events, methods, fields) are mapped to the concepts of OPC UA. The information model plays a central role as an interstation for data transferred between the network layer and the Adaptive Application.

#### 5.2.1. Events

With the existing bindings (SOME/IP, DDS), the call of the send method from the Adaptive Application triggers the transmission of a network message with event data. However, we chose a different approach for our binding. A send call from the application level only writes data into the OPC UA information model. The SourceTimestamp of the node is updated upon each write access. As AUTOSAR events are fire-and-forget notifications from the perspective of the Adaptive Application, the timestamp indicates the time at which the event was sent by the application.

**Figure 8:**
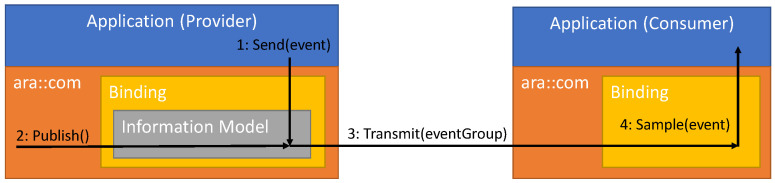
Transmission of an event from a provider to a consumer.

The actual transmission of event data are realized separately. Figure 8 depicts the publishing mechanism for OPC UA PubSub: Once the provider applications send data (1), they are stored in the information model. A separate thread triggers a publishing routine at a configured interval (2). This means that data are retrieved from the information model and assembled into a PubSub network message. Cyclic publishing takes the place of event-based publishing for a couple of reasons. First, it addresses the requirements of cyclic (hard) real-time transmission as realized with TSN TAS. Second, the OPC UA PubSub specification [26] assumes cyclic publishing as well. Third and last, cyclic publishing over PubSub allows for group events. All events of an event group are transferred inside a network message in each and every cycle. This reduces the overhead caused by protocol headers. A consumer application can detect whether an event was sent since the last cycle by comparing the included timestamps. Once the network message is ready for transmission, the publishing routine sends it to a multicast address (3). It is transmitted over a TSN-based network. Consumers that have subscribed to an event of the transmitted event group receive the message and record an event sample (4). In more detail, the binding unpacks event data and stores them in an event sample cache that is specific to the event. The Adaptive Application in the consumer can either poll the sample cache for new samples or register a callback to be notified about new samples.

As event data are stored inside the information model of an OPC UA server, clients can access them directly via C/S methodology. More specifically, clients browse through the information model of the server and read the variable node that belongs to the respective event. The client may be either a standalone OPC UA application or a part of a consumer’s network binding. Clients may also use so-called subscriptions [41]. They are part of OPC UA C/S methodology (not to be confused with PubSub). Subscriptions allow clients to subscribe to data points. The server then monitors these items and notifies clients about changes.

#### 5.2.2. Methods

AUTOSAR Adaptive methods are remote procedure calls (RPCs). A service consumer calls a method with a list of parameters and, optionally, receives a return value with the result. This concept can be mapped one-to-one to OPC UA method nodes. An OPC UA client calls a server method by passing input arguments. The OPC UA server uses a callback mechanism to run the function registered by the Adaptive Application. After completion, the server returns a list of output arguments.

Methods are only implemented for C/S access. We do not intend to realize them using OPC UA PubSub since PubSub does not support a matchable concept.

#### 5.2.3. Fields

An AUTOSAR field has, unlike an AUTOSAR event, a certain value at any time. Conceptually, it inherits features of methods and events. The value of a field can be accessed using a getter method. Writable fields contain an additional setter method. Consumers can subscribe to fields. When a field value is updated, it notifies subscribed consumers with the same mechanism as regular AUTOSAR events. We decided not to map fields in the same way. Instead, we realized fields as variable nodes in the information model. The access level of the variable node specifies whether the node can be written by a client. This substitutes the existence of getter and setter methods. Clients can subscribe to it using the OPC UA subscription mechanism. Notifications triggered by the subscription substitute the functionality that is realized as event in SOME/IP and DDS.

**Figure 9:**
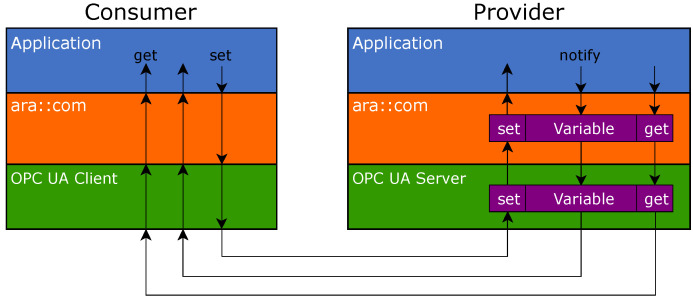
Field mapping architecture.

More technically, variable nodes for fields have an attached data source. This means that the nodes do not store the value themselves but manage callbacks that handle read and write access. Figure 9 visualizes this concept. The OPC UA server of a provider redirects every read and write request to a callback function. The particular callback function is the get handler or set handler of the binding, respectively. This handler, again, calls a get or set callback registered by the Adaptive Application. From the consumer perspective, the OPC UA client within the binding writes or reads the respective variable node inside the server information model. Event data are directly transmitted between the Adaptive Application and the OPC UA client through the ara::com interface. The OPC UA client does not have an information model.

Subscriptions regularly use sampling (polling) to detect value changes. This would lead to a performance penalty since it would trigger the get callback of the application periodically, e.g., at an interval of 50 ms. We recommend to use OPC UA’s so-called exception-based model [41] (Section 5.12.1.2) instead. This concept avoids sampling and requires the overlying logic (here: the binding) to notify the OPC UA stack about value changes proactively. The subscription then triggers a notification to subscribed counterparts.

Fields are only implemented for C/S access. We do not intend to realize them using OPC UA PubSub since PubSub does not support a matchable concept.

### 5.3. Deployment Parameters Mapping

**Figure 10:**
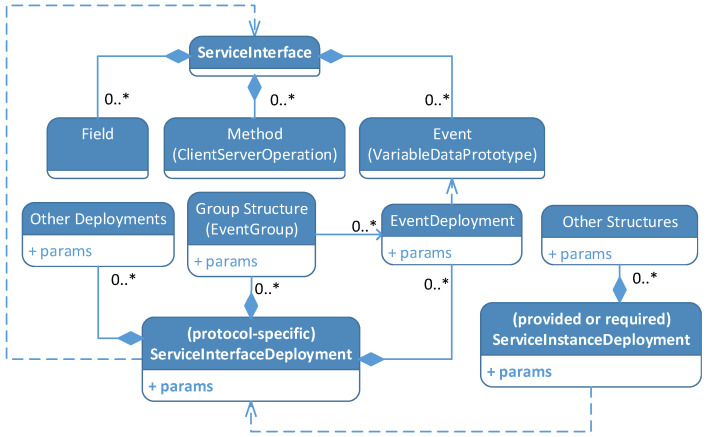
Protocol-specific ServiceInterfaceDeployment and ServiceInstanceDeployment in AUTOSAR Adaptive, see [42]. (0..*) denotes the association to none or at least one object.

AUTOSAR specifies a development methodology for Adaptive Applications. This includes a standardized approach to describe components using the AUTOSAR manifest, see Section 2.1.2. The manifest section that specifies how a service and its components are mapped to a specific communication technology is called ServiceInterfaceDeployment, see Figure 10. For example, it assigns protocol-specific numeric identifiers to services or events and provides structures and parameters to group AUTOSAR events for joint transmission. A service interface can be instantiated multiple times with ServiceInstanceDeployment. The resulting service instances are derived from a protocol-specific ServiceInterfaceDeployment. The ServiceInstanceDeployment contains parameters and concrete configurations specific to a service instance. Service instances can be again mapped to machines to obtain communication configurations in a ServiceInstanceToMachineMapping. The ServiceInstanceToMachineMapping maps e.g., multicast IP addresses to service instances and assigns communication connectors of the machine to the instance. We designed a ServiceInterfaceDeployment and a ServiceInstanceDeployment for OPC UA. Furthermore, we mapped the parameters of the deployments to concepts available in OPC UA.

**Figure 11:**
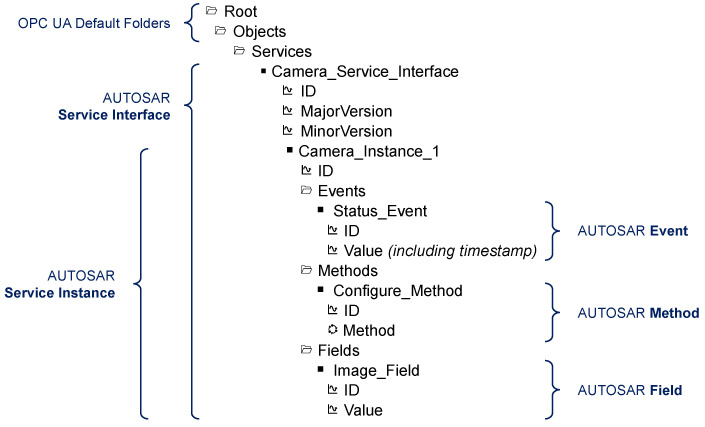
Representation of an AUTOSAR service instance in the OPC UA information model.

We have integrated the AUTOSAR service interface artifacts as a custom-designed OPC UA information model. Figure 11 shows the representation of a service instance in the information model from the perspective of a client. It is the AUTOSAR service representation of the exemplary camera sensor introduced in Figure 5. In more detail, a folder Services is placed inside the preexisting Objects folder of the OPC UA address space. The Services folder contains object nodes for every service interface offered by the provider (here: Camera_Service_Interface). Each service interface object contains OPC UA variable nodes with a numeric identifier (ID) and numeric version information (Major, Minor) of the service interface. Additionally, this object contains another object for every service instance of the respective interface (here: Camera_Instance_1). Service instance objects, again, have a numeric identifier and folders for the service components (events, methods, fields). Objects for specific service components exist inside each folder. These objects have two nodes: a numeric identifier variable (ID) and the actual content of the component (Value, Method). In this example, we represent the status of the camera as an event, the configuration routine as a method, and the image as a field.

The object nodes mentioned above are derived from custom object type definitions (ServiceInterfaceType, EventType, etc.). To promote interoperability, these definitions can later be consolidated in a specified OPC UA information model, called Companion Specification.

**Figure 12:**
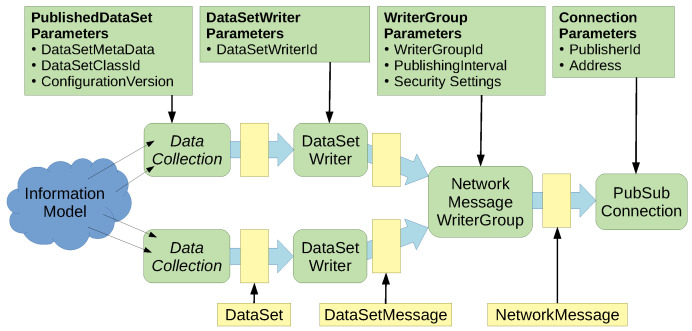
PubSub publishing sequence including configuration parameters (derived from OPC UA Part 14 [26] (Figure 6)).

The representation of AUTOSAR events in OPC UA PubSub is based on PubSub-specific configuration structures. Figure 12 shows the configuration structures involved in the PubSub publishing process, including a selection of parameters. Our mapping specifies how parameters from the AUTOSAR interface deployment are mapped to these OPC UA configuration parameters. The PubSub configuration model exposes a lot of optional configuration parameters that we did not use for our mapping approach. Particularly relevant structures are PublishedDataSet, DataSetWriter, WriterGroup, and PubSubConnection. DataSetWriters generate so-called DataSetMessages based on parameters provided in a PublishedDataSet. In our mapping, a DataSetMessage can contain the data of a single or several event samples. The DataSetMessage contains further parameters like type encoding. In more detail, the DataSetWriter processes data extracted from the information model, called DataSet in the PubSub context. DataSetWriters complement DataSets with an identifier DataSetWriterId which contains the numeric identifier of an AUTOSAR event in our mapping. As explained before, events can be grouped. This is done using WriterGroups that aggregate multiple DataSetMessages. The result is a NetworkMessage. A WriterGroup adds the WriterGroupId that we use to store an identifier of an event grouping. Furthermore, the WriterGroup is responsible for cyclic publishing. It is therefore configured with a publishing interval. The PubSubConnection is the configuration structure for the network transport. It contains the PublisherId which is a unique identifier of the publisher. We use this field to store the numeric identifiers of the AUTOSAR service interface and service instance. For example, the PublisherId has a length of 32 bits. The 16 bits interface identifier is stored in the upper half of the PublisherId, the 16 bits instance identifier in the lower half. Additionally, the PubSubConnection stores the destination address of the PubSub messages (in our case a multicast address).

**Figure 13:**
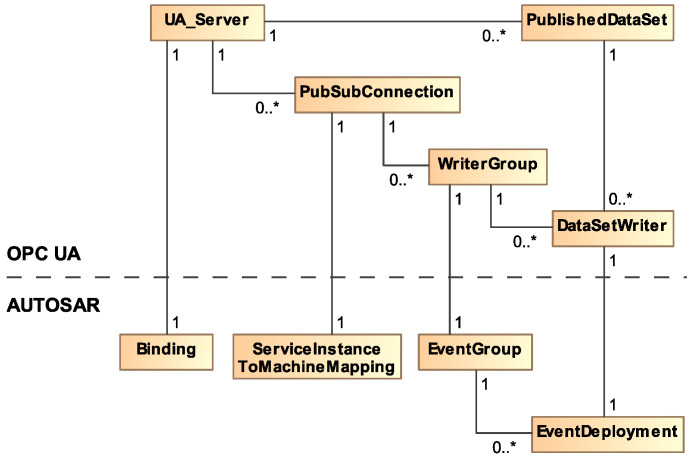
Mapping of AUTOSAR and OPC UA configuration entities for events (extension of [26] (Figure 17)). (0..*) denotes the association to none or at least one object.

Figure 13 shows a summary of the mapping described above. Configuration structures of OPC UA are mapped to AUTOSAR concepts. An OPC UA binding contains a single UA_Server. A UA_Server contains a variable number of PubSubConnections (in open62541). A PubSubConnection is created for each ServiceInstanceToMachineMapping. For every EventGroup in AUTOSAR a WriterGroup is added to the PubSubConnection. In addition, finally, a DataSetWriter is added to the WriterGroup for every corresponding EventDeployment in AUTOSAR. A PublishedDataSet is created for the parameterization of each DataSetWriter.

### 5.4. Type Mapping

Key functionality of the binding is type mapping. A consistent strategy is required to convert AUTOSAR-level data into OPC UA-compatible data that are eventually serialized into an on-wire byte stream. The consumer side must have the same understanding of data types to properly deserialize the byte stream and convert it into high-level AUTOSAR-compatible data. For this purpose, OPC UA provides a comprehensive data modeling framework that can be exploited in different ways.

AUTOSAR supports basic types (Integer, Float, etc.) as well as structured types which are compositions of basic types. A general constraint is that instances of these structured types must be written in one transaction, i.e., as a whole. It must not be possible to modify single structure elements separately. Therefore, it is not possible to map structure elements to separate OPC UA variable nodes. The following sections propose two options for type mapping. The first option is based on OPC UA container type variant. The second option makes use of custom data types.

#### 5.4.1. Variant-Based Mapping

Variant-based mapping makes only minimal use of OPC UA’s rich data modeling. This strategy is based on variants, a generic container type. Variants can hold any other data type as scalar or as an array [43]. This facilitates to map structured data types in AUTOSAR to nested variant arrays in OPC UA. It follows the paradigm “Everything is in a variant”.

The variant-based mapping completely decouples providers and consumers on OPC UA level. No explicit exchange of OPC UA data types is necessary. As a result, this approach does not ensure type safety on an OPC UA level. The OPC UA stack only processes the variants and passes data values to the higher level AUTOSAR logic. Type safety and information interpretation are realized on an AUTOSAR level. Therefore, applications that do not run on top of AUTOSAR must perform type binding on application level.

#### 5.4.2. Custom Data Type Mapping

This option especially covers the use case of communication between AUTOSAR and non-AUTOSAR applications. OPC UA applicants regularly make use of custom type modeling to represent complex data structures in the address space. That raises the need for the binding to support type mapping based on custom type definitions. The usage of custom types must be fully transparent to any AUTOSAR application.

In more detail, the OPC UA information model contains a set of predefined data types. Among them is the subtype Structure, which, again, contains subtypes. Model designers can create custom subtypes of Structure. The subtypes have an attribute of type StructureDefinition. This attribute contains (among other values) an array of elements that describe the single structure elements. OPC UA transfers values of Structure data types in a byte string. To decode the byte string, the information defined in the StructureDefinition is needed.

Several options exist for the exchange of data type definitions. They differ in the time of exchange. Consumer applications can receive type definitions at compile-, startup-, or at runtime. Compile-time exchange is realized by compiling and linking source files with type definitions into consumer applications. When using startup-time exchange, the consumer reads a binary file containing type definitions. Runtime type exchange can be realized with C/S communication between consumer and provider. Setting up a central directory for type definitions is another option. In [44], we are presenting details about type exchange options.

### 5.5. Service Discovery Mapping

As its name already suggests, AUTOSAR Adaptive focuses on the flexible and dynamic interaction between vehicle components. Service consumers are capable to find matching service providers. The Adaptive Platform allows communication path establishment at design-, startup-, or runtime. For the two latter approaches, a central service registry is specified in the AUTOSAR standard. The registry implements three discovery operations: Service providers announce their service with Offer and withdraw it using StopOffer. Subscriber applications query the registry for a specific Service by calling Find. The registry concept itself is abstract and protocol-agnostic. It must be mapped to a specific protocol.

We evaluated OPC UA discovery mechanisms for usage as service registry. OPC UA specifies two discovery mechanisms: Local Discovery Server (LDS) and Global Discovery Server (GDS). The LDS keeps track of OPC UA servers running on a host system and exposes them publicly. The GDS, on the other hand, can be used for discovery across multiple subnets. Since intersubnet discovery may be required by certain use cases, we chose the GDS for the role as registry. A GDS exposes an information model that contains UA methods for registration and query of OPC UA applications [45].

Since not all properties of an OPC UA application match the properties of an AUTOSAR service, we designed an explicit mapping of service properties to properties of OPC UA applications. The GDS provides certificate management services, too. This capability may be exploited for encryption and authentication.

In a later phase of our research, we found that some requirements cannot be fully met with a GDS-based service registry. Two major shortcomings had an impact. First, the GDS does not implement a concept that corresponds with the TTL (time to live) of an AUTOSAR service. Second, the GDS does not allow domain specific extensions. This means that additional domain specific attributes cannot be attached to an application record. For these reasons, we decided to come forward with a proposal for an alternative registry concept. This custom service registry is a domain specific OPC UA server. In the binding specification, we describe an information model with data types (e.g., ServiceRecordDataType) and methods (e.g., OfferService). Both directly map the AUTOSAR concepts.

Beside these centralized discovery approaches, a distributed option is introduced in the AUTOSAR standard [46]. With the distributed approach, a single responsible registry is not needed. The registry information is distributed among participants via broadcast communication. Applications hold a self-administered view of the registry. We are seeking to implement the distributed discovery using OPC UA PubSub in the future. Therefore, we added data types to the custom service registry that represent registry operations (i.e., Offer, StopOffer, Find). Instances of the types are transmitted inside PubSub messages. While being specific about data types, we left the publisher configuration (e.g., WriterGroups, DataSetWriters) unspecified. If a domain with a single responsible registry and a domain with distributed registry need to be connected, a discovery bridge can link these domains [46]. The discovery bridge also holds a local registry and acts as a broker between the domains.

## 6. Binding Implementation for Real-Time Systems

We developed the OPC UA integration (binding) based on the AUTOSAR reference implementation version 19-03. It is written in C++. With respect to OPC UA, we decided to use the open-source implementation open62541. It is written in the C programming language. Its source code is available on GitHub. We used the GDS implementation that is available with .NET Standard Stack from the OPC Foundation (https://github.com/OPCFoundation/UA-.NETStandard-Samples/tree/master/Samples/GDS, last accessed on 1 March 2021). Microsoft’s .NET Core allows running .NET applications on diverse platforms, including Linux. The following subsections explain noteworthy aspects of the implementation.

### 6.1. Multithreading Support

For the sake of responsiveness and execution speed, the AUTOSAR platform uses multithreading. In general, real-time systems must implement measures to guarantee integrity and determinism in multithreading scenarios. For this purpose, open62541 introduces different levels of multithreading support. With activated multithreading support, API calls (e.g., UA_Server_read and UA_Server_write) are mutually exclusive. This means that a write operation cannot be preempted by a read operation. For a deterministic PubSub-only publisher, we assume two threads: a publishing thread with read access to the information model and an application thread with write access to it. The time-critical functionality of both threads would be mutually exclusive. One thread would have to wait for the completion of the other. For determinism, both threads must have reentrant (i.e., lock-free) information model access. Hence, the aforementioned multithreading mechanisms are inapplicable.

Contributors of the open62541 project propose a lock-free mechanism that guarantees a consistent view on the information model and data integrity. What they describe in [16] is a copy-and-replace technique: The underlying data structure of the information model is a hash map pointing from the node identifier to the node representation in memory. Each node is treated as immutable, i.e., it cannot be modified once it has been inserted into the hash map. For modifications, it is possible to replace the node with a modified copy. The replacement is performed using an atomic compare-and-swap (CAS) operation. This way, the information is always consistent, even if a write operation is interrupted mid-update. Information model access can be reentrant. The implementation of this mechanism comes with the open62541 major branch.

The binding implementation uses this concept. However, since the binding is a complex scenario that requires deterministic behavior in a multithreading environment, it requires further measures. In the following subsection about real-time memory management, we build upon this concept and extend it.

### 6.2. Real-Time Memory Management

The implementations of the Adaptive Platform and open62541 make recurring use of dynamic memory management, i.e., heap allocations with malloc/free in C or new/delete in C++. The paper [47] points out the following problems with regular dynamic memory management: sufficiency (refusal of allocation), memory leaks, fragmentation, and timeliness (undefined timing behavior).

For these reasons, it is highly discouraged to use dynamic memory management in real-time systems requiring deterministic behavior. Some embedded systems avoid the use of dynamic memory management, and thus the heap, altogether [48]. However, both the Adaptive Platform and OPC UA are in nature dynamic. AUTOSAR Adaptive requires dynamics for its scalability. OPC UA information model has a database character. Hence, it is not possible to eliminate dynamic memory management in the whole application.

**Figure 14:**
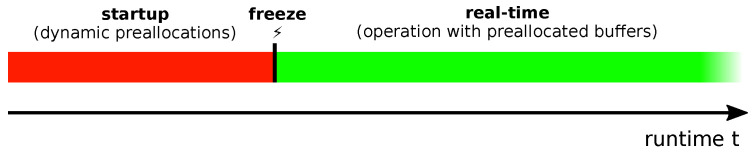
Real time application lifecycle.

We tackled this problem by eliminating dynamic allocations only in time-sensitive paths. As Figure 14 shows, dynamic memory management is restricted to the system startup phase. Buffers that are needed for real-time operation are preallocated. A freeze event introduces the real-time phase in which the buffers are used.

In more detail, the complete program path reaching from the platform method for sending an event to the actual PubSub message generation logic must be examined. Timely generation of network messages is especially crucial in conjunction with TAS setups with cyclic transmission of real-time data. If a packet is not ready to be sent when a cycle starts, it must wait until the next cycle.

In a first step, we replaced all dynamic containers (like the STL container vector) with preallocated custom containers. Using a standard STL container with a custom allocator, as shown in [49], would have been possible as well.

In a second step, we extended the concept of immutable nodes described above. It is problematic that a new node is allocated upon each write access to a node. We decided to preallocate two buffers for each node that stores event data. Upon node modification, the node currently referenced by the hash map is copied to the other buffer. Once the modification is complete, the reference in the hash map is changed to the other buffer (buffer alternation).

In the last step, we handled the remaining dynamic allocations in the message generation functionality. It is beneficial that all memory allocation during the generation of a PubSub message is freed after the message is sent. Thus, we could redirect all allocations within the message generation to a preallocated buffer. Knowing that none of the allocated memory is needed after sending the message, the subsequent publishing routine can write into the buffer again starting at address 0.

An alternative to the restriction of dynamic memory management is the usage of a real-time memory allocator. Such allocators pursue to overcome the variable runtime and fragmentation inherent to regular allocators. One example is the TLSF allocator described in [50]. Allocations have constant runtime, i.e., O(1). A drawback is that current implementations do not support thread safety. It must be provided by the integrator.

### 6.3. System Level Synchronization

**Figure 15:**
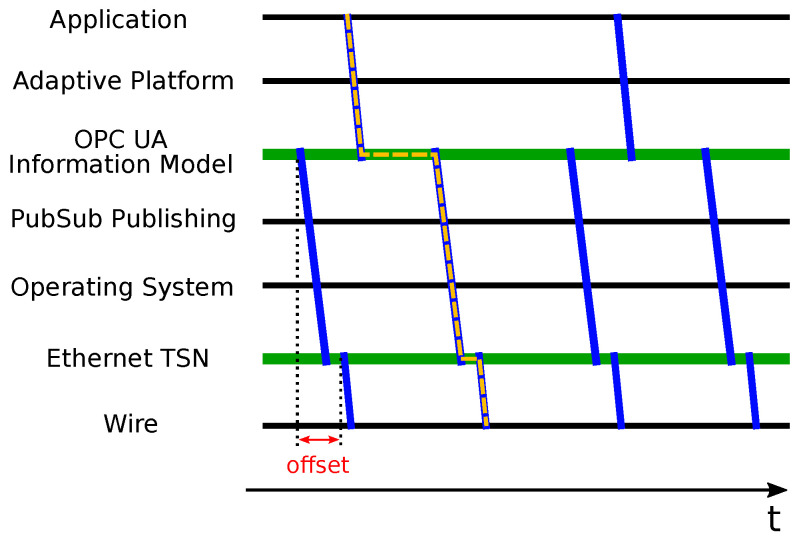
Information propagation through system levels.

Event data generated on the application level of a service provider have to pass through different system levels until it is finally transmitted on wire. Figure 15 shows these different levels and data flows (blue lines). The yellow dashed line visualizes the data flow of an event: Data are sent from the application level to the OPC UA information model using methods of the Adaptive Platform (specifically ara::com). The information model is a buffering stage that holds data in nodes. The separate publisher thread accesses the nodes and assembles a network message that is sent through an operating system socket. The Ethernet TSN network interface, again, is a buffering instance that holds packets until the start of the assigned time slot (see Section 2.3).

At the moment, scheduled cyclic execution of Adaptive Applications is not yet realized as platform functionality. For this reason, we used oversampling on the publishing level in order to reduce event sample loss. This means that the publishing interval is shorter than the assumed minimal send interval of the application. Figure 15 shows a factor 2 oversampling, i.e., the publishing is triggered twice as often as the application sends data.

The TSN adapter and the application space must have a common notion of time. This is achieved by synchronizing a system clock (here: CLOCK_REALTIME) to the adapter’s PTP Hardware Clock (PHC), which itself was synchronized with the PHC of other TSN participants. It is thereby possible to start the generation of PubSub messages at a specified time relative to the TSN cycle start (offset). This method cannot only guarantee that a network message is ready for transmission upon cycle start, but it also ensures that the data are up-to-date. The cyclic publishing is technically realized within a dedicated thread. Its core is a loop that in each iteration waits for the next scheduled cycle (using POSIX method clock_nanosleep), executes the publishing, and calculates the next wakeup time.

It is also possible to introduce a second offset that synchronizes the application-level data provisioning and the OPC UA publishing. The Adaptive Applications sends event data at a specified time before the publishing routine reads data from the information model. One way to realize this is by using the POSIX method clock_nanosleep in the implementation of an AUTOSAR application. It should be noted that this behavior is not part of the AUTOSAR platform. It must be implemented by the application programmer.

### 6.4. Real-Time Scheduling Considerations

To achieve system-wide real time, deterministic network transmission is not the only requirement. Holistic system design must also cover determinism on the application level. In the AUTOSAR environment, Execution Management (see Section 2.1.2) allows the configuration of scheduling and resources. It works together with the operating system to initialize the runtime scheduling of applications [5]. The Deterministic Client, as introduced in the Execution Management Specification [21], is supposed to support the event and time-triggered execution of Adaptive Applications in the future. It offers a deterministic worker pool, distribution of activation time stamps to Adaptive Applications, and random numbers generation [21]. Not all details about the Deterministic Client are given yet. EM is not responsible for the performance of the scheduling. It is the responsibility of the operating system. In order to provide real-time resources to applications, the person or the tool integrating applications into platforms and machines is in charge of allocating enough resources, assigning appropriate scheduling policies and priorities, and monitoring deadlines. The configuration of resources using the support of the Execution Management can be specified in the AUTOSAR manifest. In this case, scheduling parameters in the manifest are based on the operating system interface POSIX (IEEE 1003.1 [51]). Real-time scheduling policies SCHED_FIFO (First In, First Out) and SCHED_RR (Round Robin), as well as non-real-time policy SCHED_OTHER can be selected as valid scheduling policies for applications using EM [21]. Other scheduling policies such as SCHED_DEADLINE are not prohibited, but they may not be portable across different AP implementations [5]. The scheduling policy SCHED_FIFO allows realizing scheduling algorithms such as the rate-monotonic scheduling with fixed priority assignments [52] (p. 354).

One prerequisite is the reliable determination of the worst-case execution time (WCET) of an application that is an important metric for schedule calculation. The operating system strongly affects the WCET as it introduces task scheduling overhead, interferences between running tasks, and system call execution. Unfortunately, in general-purpose systems with real-time extension (e.g., Real-Time Linux), the upper bound of the WCET is difficult to estimate and may be too pessimistic compared to special-purpose embedded systems [53].

Another prerequisite is the ability to preempt the execution of threads, e.g., as provided in the Linux PREEMPT_RT patch (https://wiki.linuxfoundation.org/realtime/start, last accessed on 18 March 2021). High priority threads must be able to preempt running low priority threads. The fixed assignment of priorities complicates the addition of new threads with latency bounds at runtime. As a result, the old schedule may become invalid and applications could miss their deadlines. Thus, all applications demanding tight latency bounds should be known and considered before runtime to achieve an optimal or near-optimal schedule.

As AP promotes flexibility and scalability, distribution of applications across different AUTOSAR platforms and machines is possible. For distributed cyclic real-time applications, it means an increased complexity to create a feasible schedule to satisfy all end-to-end latency requirements. The end-to-end latency of communicating applications comprises the execution of the provider applications, the transmission of the messages over IPC or a TSN network in our case, and the execution of the consumer applications. If the network is involved, both the network and the task schedules have to be coordinated. One solution to address task and TSN network scheduling has already been investigated in [54]. In future works, we will elaborate on the subject matter in more detail.

### 6.5. TSN Configuration and Parameter Mapping

During our research, the OPC Foundation was working on a specification for a configurable transport mapping of OPC UA PubSub to TSN. The specification was in draft state and not publicly available. This means that a generic mapping solution as it would be useful for the AUTOSAR network binding was not yet available. The configuration of TSN (TAS) must be done under consideration of the holistic system design, anyway. This includes the definition of streams for different service classes, e.g., real-time or best-effort. TSN scheduling configuration is a field of its own that is currently under investigation, as discussed in our previous work [29]. Specific capabilities of the employed TSN devices must be taken into account as well. Temporarily, we set the TSN configuration manually. This includes the setup of time synchronization (e.g., PTP), VLAN configuration (VLAN ID and priority mapping) as needed by IEEE 802.1Qbv and the installation of a TSN schedule (cycles, slots).

## 7. Performance Evaluation and Results

In this section, we evaluate the performance of the implemented communication scheme. The evaluation is a proof of concept to show that the combination of the three technologies is beneficial for future automotive systems. This includes a brief analysis of the memory consumption of a provider and a detailed analysis of the timing behavior of the service provider.

### 7.1. Memory Analysis

To evaluate the readiness of this scheme for low-end IoT devices, we measured the RAM usage of a sample application. We ran the test on a VM with Ubuntu 18.04.3 LTS. Version 1.0 of the open62541 stack was statically linked to the AUTOSAR platform (i.e., one single binary). The AUTOSAR test application is a small provider that publishes 30 Byte of payload periodically. We used the Linux tool top for the measurement. The results show that the AUTOSAR provider including open62541 consumes 6.3 MB. By measuring the memory usage of a standalone OPC UA instance with the same properties, we found that the OPC UA middleware consumes approximately 40% of the memory of the whole AUTOSAR instance. It must be noted, however, that the memory consumption of an AUTOSAR application heavily depends on the platform functionality used as well as deployment and linking aspects. The generalizability of this measurement is strongly limited.

### 7.2. Timing Analysis

Accurate and reliable timing behavior is a crucial feature of critical real-time systems. After introducing the setup for our timing analysis, we examine the performance of the TSN link layer and the timeliness of the publishing routine as intermediate steps. Finally, we present the observed latencies of an end-to-end scenario. This timing analysis only focuses on the evaluation of event transmission via OPC UA PubSub. We do not evaluate the timing behavior of the OPC UA C/S communication as there are no stringent determinism requirements for C/S best-effort communication. However, we successfully tested the functionality of the C/S mapping (events, methods, fields) with an information model similar to the example in Figure 11. The PubSub event data are retrieved from the same information model as used in the OPC UA C/S mapping.

#### 7.2.1. Setup

**Figure 16:**
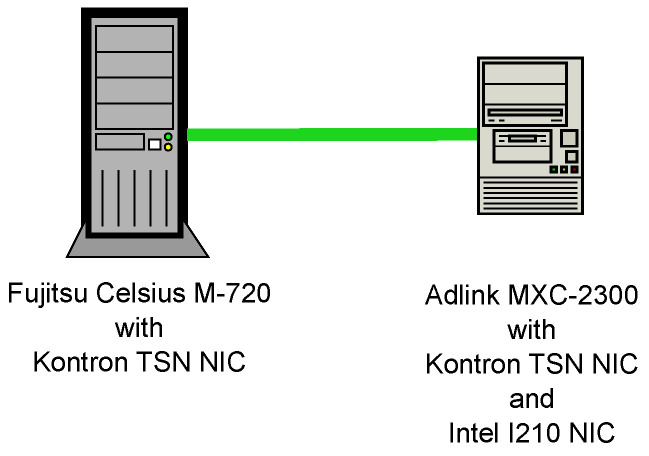
Test setup for the timing analysis.

As shown in Figure 16, our test setup involves two entities: A Fujitsu (Tokyo, Japan) desktop computer acts as the provider (Celsius M-720, Intel Xeon CPU E5-1620, 4 cores, 3.60 GHz). An embedded computer from Adlink (Taipei, Taiwan) is the subscriber (MXC-2300-3E1(G), Intel Atom E3845 processor, 4 cores, 1.91 GHz). We additionally equipped both devices with the network interface card Kontron PCIE-0400-TSN that supports among others the TSN standards IEEE 802.1AS and IEEE 802.1Qbv to enable time-triggered injection of OPC UA frames. Provider and consumer are connected end-to-end over a 1 Gigabit Ethernet physical layer.

We equipped the CentOS operating system with a Linux real-time kernel (4.18.0-80.11.2.rt9.157.el8.x86_64). It is a standard (“vanilla”) kernel that received real-time capabilities through the patch PREEMPT_RT. To achieve determinism, it replaces locking primitives with preemptive implementations, introduces priority inheritance and converts interrupt service routines into threads [55]. The patch enables the usage of real-time scheduling policies (Round Robin, FIFO) and priorities for threads. Timing analysis of PREEMPT_RT and further explanations can be found in [56].

#### 7.2.2. Link Layer Timing

Since the proper operation of the TSN link layer is crucial to the following tests, we briefly introduce steps to configure and test the precision of TSN TAS with the setup described above.

It is advisable to run TSN configuration steps automatically, e.g., with a shell script. The script starts with the synchronization of PTP hardware clocks of the network interfaces with the tool ptp4l. To optimize the system’s timing behavior, the script instructs all CPUs to run at maximum frequency. Another timing tweak is to configure the IRQ affinity so that the interruption of the TSN network interface is processed by a single CPU core. Then, Linux’s traffic control is configured with the queuing discipline FIFO (tc qdisc). In a next step, the script adds a VLAN interface and sets egress map of the new VLAN device (mapping socket buffer priority to VLAN priority PCP) using vconfig. Finally, the TSN schedule is set with the custom tsntool.

We used the tool netlatency (https://github.com/kontron/netlatency, last accessed on 1 March 2021) to measure the latency and jitter characteristics of the end-to-end connection. The provider side generates UDP frames at an interval that is synchronized with the TSN cycles of 1 ms. Various timestamps are recorded and written into the frames. For example, timestamps are recorded before the packet enters the Linux packet scheduler, before the packet is passed to the network interface, and upon reception in the interface of the remote device. This allows for tracing the timing behavior of different stages within the process. Frames are sent through the Kontron interface with the configured schedule. They are assigned a high-priority TAS slot that starts 50 μs after the cycle start and is open for 100 μs. The consumer side captures the frames through the I210 interface, adds receive timestamp information, and saves the results. After a specified count of captured frames (here: 10,000), the tool generates and prints statistics. We used the packet generator Ostinato (https://ostinato.org/, last accessed on 1 March 2021) to simulate network load. The tool flooded the interface with bulk load which was scheduled as best-effort traffic. We verified the arrival of bulk packets at the remote station with the Linux tool tcpdump. The output of netlatency showed that all timestamp frames arrived at the remote station with a maximum jitter of 1 μs. The reason for this low jitter is that packets were prevented from sending by the closed TAS gate before the actual beginning of the high-priority slot, even if they arrived too early at the hardware egress queue of the network interface card. The worst-case latency from the start of the interval on the sending device until the reception on the remote device was 51 μs. The overhead caused by the packet scheduler and the kernel driver, i.e., the latency measured from sending the frame by the netlatency application until the actual enqueuing in the network interface, is less than or equal to 25 μs, see Table 1. The link layer evaluation proves that TSN TAS complies with the scheduled times. However, this evaluation shows that we have to consider a small overhead caused by the packet scheduler and the kernel driver when synchronizing and scheduling real-time applications. We point out that this measurement depends, among others, on the used hardware and the length of the transmitted message.

#### 7.2.3. Publishing Timeliness

When using TAS, it is crucial that packets with time-sensitive content are ready for transmission when their dedicated timeslot starts (see Figure 15). More specifically, the binding must pass event data messages to the underlying TSN (TAS) device reliably on time.

We first analyzed the timeliness of the provider’s publishing using OPC UA PubSub. This is the time between the scheduled start of the publishing and completion of the publishing routine. The handover of the PubSub message to the operating system network stack (socket access) is included. Neither the behavior of the provider application nor the message handling in a remote consumer was considered. The publishing thread was started with the SCHED_FIFO policy and the highest priority (99). Assigning a thread to a single CPU core reduces jitter. To simulate CPU load, we used the Linux tool stress which spawned 10 threads performing dynamic memory allocations.

The final results show that the publishing latencies in a load scenario are distributed in a compact cluster with a maximum latency of 94 μs, see Table 1. For the following AUTOSAR test, we allow 120 μs for the publishing. This is the maximum latency measured in the stress scenario plus a safety gap (e.g., for packet scheduling overhead). It represents the offset shown in Figure 15.

#### 7.2.4. End-to-End Latency

Based on this insight, we extended the test and realized an end-to-end scenario: One AUTOSAR provider application sends event data with the current timestamp (and redundant copies) to the consumer application. The clocks of both network interfaces are synchronized with the Linux tool ptp4l. A system clock is synchronized to the PTP hardware clock of the interface using phc2sys. Hence, the transmission time can be determined in the consumer by calculating the timestamp difference on application level. This end-to-end measurement covers a comprehensive set of steps that make up the system timing behavior. It includes the link layer timing and the publishing timeliness outlined above. We chose this evaluation method because it is close to real-world scenarios, yet simple enough to extract clean performance figures.

**Figure 17:**
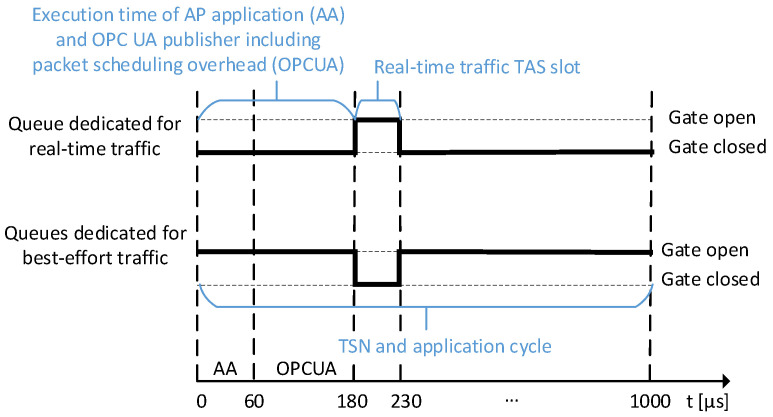
Time-Sensitive Networking and application cycle of the AUTOSAR provider.

In more detail, we first configured a TSN schedule that reserves a 50 μs slot for critical traffic within a 1 ms cycle, see Figure 17. The slot must be sufficiently long for the content to be transmitted and some additional jitter, e.g., caused by time synchronization deviation and hardware. For the consumer side, we wrote two applications. The first is a simple OPC UA-only application that processes incoming timestamp data to show the compliance of our binding with existing OPC UA implementations. We pursued to process messages as straightforwardly as possible. Its message processing logic is completely free of dynamic allocations. The second is an AUTOSAR instance that uses the Standard Template Library (STL) container deque inside the binding implementation to buffer incoming events. This container performs dynamic allocations regularly.

A measuring cycle is scheduled to begin 60 μs before the start of the publishing (see Figure 17). This is the longest measured duration for this operation ( 46 μs) plus a safety buffer. Once the application thread wakes up (return of Linux method clock_nanosleep), the provider application retrieves the current timestamp and sends it wrapped into an event. The publishing is scheduled 120 μs before the start of the high-priority TSN slot. (To calculate a more precise schedule, other delays such as context switching by the operating system must be considered.) It retrieves previously sent event data from the information model and sends the network message through the network stack of the OS. By starting the AUTOSAR application and the publishing sufficiently long before the gate of the real-time egress queue opens, it is ensured that a message will not miss its slot. In case of a missed slot, a cyclic message would have to wait until the next cycle. Once the message (135 Byte) reaches the remote station, the high priority consumer application captures a timestamp itself and calculates the difference to the timestamp in the received packet. This is the total end-to-end transmission time.

**Figure 18:**
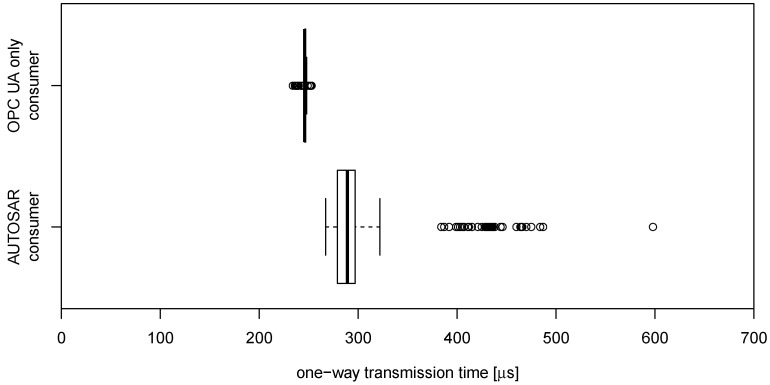
Distribution of end-to-end transmission times between a provider and a consumer application.

Our requirement was to keep the end-to-end latency less than or equal to the cycle times. We tested our binding with an application and TSN cycle of 1 ms. Based on 10,000 measuring cycles, the maximum end-to-end latency measured with OPC UA-only consumer was 253 μs (see Figure 18). Latencies are distributed over a 19 μs interval. For the AUTOSAR consumer, a substantially higher maximum latency of 598 μs was caused by an outlier. This result shows that the usage of STL containers with dynamic memory allocations prevents compliance with stringent determinism requirements. The results also show that the provider did not miss a TSN time slot and that the TAS schedule regulated the occurring jitter. Every slot was filled with exactly one packet.

Additionally, we verified the robustness of the setup with an endurance test of eight consecutive hours (28,800,000 messages). Again, the provider did not miss a time slot. Our changes in the information model memory management (alternating buffers for nodes) led us to test data integrity. By comparing the redundant timestamp copies within the event samples, we found that no sample was corrupt.

Generally, the performance expectation of an AUTOSAR provider with OPC UA middleware and TSN can be derived from existing evaluations of OPC UA over TSN. This is due to the fact that in this case AUTOSAR only acts as a thin wrapper with little influence on the overall timing. The paper [16] shows, for example, that ultra-low latencies can be achieved at a cycle time of 100 μs. However, the application WCET is a limiting factor for the cycle time if end-to-end latency takes application execution and the transmission of messages into account. In practice, several real-time applications will share the same computing, network, and memory resources. For example, ADAS functions are complex and resource-intensive. Thus, further and more detailed analyses on resource scheduling and allocation have to be carried out. The main purpose of the timing analysis is to show that real-time communication over AP, OPC UA PubSub, and TSN can be realized with a certain configuration and scheduling effort. The current AUTOSAR Adaptive Platform development pursues to simplify this effort.

## 8. Binding Architecture Validation

Based on our experiences and the measurements described above, we have evaluated the proposed software architecture on the basis of common criteria for software quality characteristics described in ISO 9126 [57], see Table 2. We found that interoperability and scalability are strong advantages of the platform. Adaptability and conformance with existing specifications open the way for simplified customizability and portability. Simple reuse of software components is facilitated by AUTOSAR Adaptive. These aspects have the potential to reduce development and maintenance costs. The memory efficiency allows operation on devices that are commonly used in vehicle automation. However, the runtime efficiency of the proposed architecture heavily depends on the underlying operating system and its process scheduling. A misconfigured or overstrained scheduler can impair overall system dependability. In addition, the architecture lacks maturity as the development of all involved technologies is still ongoing. Nonetheless, the non-satisfied characteristics are currently being addressed by the AUTOSAR development partnership, the OPC Foundation, and the IEEE 802.1 TSN Task Group.

## 9. Conclusions

By proposing an integration approach of the AUTOSAR Adaptive Platform, OPC UA, and TSN, we presented a holistic solution for modern vehicle communication. Particularly, we showed that a combination of OPC UA C/S and PubSub qualifies as middleware for the Adaptive Platform. Our main contribution is the binding concept between the Adaptive Platform and OPC UA that harmonized the architectures of both technologies. We proved the concepts with a prototype and presented notable tweaks of the implementation. Furthermore, we used this implementation to deploy a TSN test setup and verified a deterministic communication schema in an end-to-end scenario. However, as our evaluation shows, the scheduling effort of AP applications, OPC UA PubSub, and TSN should not be underestimated.

The merit of the AUTOSAR Adaptive platform is its development methodology that enables efficient software engineering. By using OPC UA as a communication protocol, the Adaptive platform adopts a widespread standard that provides a rich information and communication model. This allows manufacturers to purchase commercial off-the-shelf components and avoids vendor lock-in. Similarly, TSN is expected to be implemented by network components that are available in a competitive market. The convergence of real-time and best-effort traffic in a single physical network saves costs and weight. However, further work needs to be done to promote determinism in Adaptive Applications. Time and data determinism must be guaranteed using elaborate resource management and scheduling. The AUTOSAR Deterministic Client [21] aims to address these requirements.

In addition, the TSN standardization is still in progress. Thus, there is still a lack of appropriate and ready-to-use TSN hardware to realize reliable automotive systems. Furthermore, the configuration of TSN-capable devices has not yet been fully investigated. The configuration aspect of TSN devices will not be part of the TSN standardization [35]. There is especially a need for concepts to configure end-systems. However, some papers have already addressed this problem by proposing OPC UA as a possible solution [32,37,38].

In the long run, we pursue the integration of our binding specification into the official AUTOSAR Adaptive release alongside existing network bindings. This step would address the rising relevance of OPC UA and TSN across domains.

## Figures and Tables

**Table 1:** Worst-case (WC) measured latencies on different levels of the publishing process.

Level	Description	WC Latency
AP App	Duration of the AP application that processes and prepares the data to be transmitted.	46 μs
PubSub App	Duration of the OPC UA publishing application that wraps up the data prepared by the AP app in an OPC UA NetworkMessage before forwarding it to the network interface card.	94 μs
TSN Sched	Latency caused by the network scheduler and kernel driver after calling the send method by the publishing application and before enqueuing in the network interface card.	25 μs

**Table 2:** Compliance of the proposed architecture with quality characteristics. (✓: mostly or fully compliant, (✓): partially compliant, ✗: not compliant).

Characteristic	Evaluation	Annotation
Interoperability	✓	The architecture enables communication with other AP instances and with OPC UA-only communication partners.
Security	✓	AP and OPC UA provide a wide range of security features. TSN offers filtering and policing methods in IEEE 802.1Qci.
Time Behavior	(✓)	Our evaluation example shows that ultra-low end-to-end latency (<1 ms) is achievable in stress situations. However, more extensive tests have to be performed to examine the behavior of the OS and the AP more closely.
Memory Efficiency	✓	Memory requirements can be met with devices that are commonly used in vehicle automation.
Scalability	✓	The Publish–Subscribe pattern realized by OPC UA PubSub promotes scalability inside a vehicle network.
Operability	(✓)	The user can operate and control each technology of the architecture. AP can be configured and parameterized using AUTOSAR manifests. OPC UA is configurable over the information model. TSN in end-systems is configurable over tools like tsntool. However, the scheduling of applications and network streams has to be done by the user at the moment.
Understandability	✓	AP, OPC UA, and TSN are well documented in code, standards, and dedicated documentation.
Adaptability	(✓)	The proposed architecture can be adapted to new environments by adjusting AUTOSAR manifests, OPC UA configuration over the information model, and TSN configurations using TSN tools. However, network and application scheduling are complex tasks that need to be done separately for every system setup.
Conformance	✓	The technologies used in our architecture are compliant with AUTOSAR, OPC UA, and TSN specifications.
Maturity	✗	All involved technologies are still in development and are not yet mature.
Fault Tolerance	(✓)	Our endurance test showed that all values arrived on time and no value was lost. However, the generalizability of this measurement is naturally limited to the specific setup.

## Data Availability

The data presented in this study are available on request from the corresponding author. They are restricted to experimental results. The data are not publicly available due to third party restrictions.

## Sample Availability

Samples of the performance evaluation are available from the authors.

## Abbreviations

The following abbreviations are used in this manuscript:

AA	Adaptive Application
AD	Automated Driving
ADAS	Advanced Driver Assistance Systems
ADF	Automated Driving Function
AMQP	Advanced Message Queuing Protocol
AP	AUTOSAR Adaptive Platform
ARA	AUTOSAR Runtime for Adaptive Applications
AUTOSAR	Automotive Open System Architecture
C/S	Client–Server
CAS	Compare-and-Swap
CM	Communication Management
CP	AUTOSAR Classic Platform
DDS	Data Distribution Service
ECU	Electronic Control Unit
EM	Execution Management
FLC	Field Level Communications
GDS	Global Discovery Server
HTTP	Hypertext Transfer Protocol
HTTPS	Hypertext Transfer Protocol Secure
IEEE	Institute of Electrical and Electronics Engineers
IIoT	Industrial Internet of Things
IP	Internet Protocol
IPC	Interprocess Communication
JSON	JavaScript Object Notation
LDS	Local Discovery Server
M2M	Machine-to-Machine
MDPI	Multidisciplinary Digital Publishing Institute
MQTT	Message Queuing Telemetry Transport
OPC UA	Open Platform Communications Unified Architecture
OS	Operating System
OSI	Open Systems Interconnection
PHC	PTP Hardware Clock
POSIX	Portable Operating System Interface
PTP	Precision Time Protocol
PubSub	Publish–Subscribe
QoS	Quality-of-Service
ROS	Robot Operating System
RPC	Remote Procedure Call
SOA	Service-Oriented Architecture
SOME/IP	Scalable Service-Oriented Middleware over Internet Protocol
STL	Standard Template Library
TAS	Time-Aware Shaper
TCMS	Train Control and Management Systems
TCP	Transmission Control Protocol
TLS	Transport Layer Security
TSN	Time-Sensitive Networking
TTL	Time to Live
UADP	Unified Architecture Datagram Protocol
UDP	User Datagram Protocol
WCET	Worst-Case Execution Time
XML	Extensible Markup Language
